# Expression of Zeb1 in the differentiation of mouse embryonic stem cell

**DOI:** 10.1515/biol-2022-0042

**Published:** 2022-05-09

**Authors:** Ting Chen, Peng Pan, Wei Wei, Yanmin Zhang, Guanghui Cui, Zhendong Yu, Xin Guo

**Affiliations:** Guangdong and Shenzhen Key Laboratory of Male Reproductive Medicine and Genetics, Peking University Shenzhen Hospital, 1120 Lianhua Road, Shenzhen, Guangdong 518036, China; Department of Pathology, Children’s Hospital of Soochow University, Jiangsu, 215300, China; Department of Pathology, Guangzhou Medical University, Guangzhou, 510182, China; Department of Blood Vessel Surgical Treatment Area, Changchun Provincial People’s Hospital, 1183 Industrial and Agricultural Road, Changchun, 130021, China

**Keywords:** Zeb1, Lin28A, embryonic stem cells, mouse embryonal carcinoma cells

## Abstract

Embryonic stem cells (ESCs) differentiation is a process of replication and refinement, and the directional lineage differentiation of ESCs involves the epithelial-mesenchymal transition (EMT)- mesenchymal-epithelial transition (MET) process. A previous study revealed that Zinc finger E-box-binding homeobox 1 (Zeb1) plays a vital role in EMT, which could repress E-cadherin promoter and induce an EMT in cells. To verify the expression of Zeb1 and its correlation with Lin28a in mouse ESCs differentiation, we performed qRT-PCR and western blots to detect the expression of Lin28a mRNA and protein after Zeb1 knockdown. The expression of Zeb1 decreased over time of mouse ESCs differentiation but significantly increased in mouse embryonal carcinoma cells. After knockdown of Zeb1, Lin28a and Vimentin expression were decreased, while E-cadherin expression increased both in mouse ESCs, EBs, GC1, and P19 cells. We found that Zeb1 promoted the invasive ability of mouse embryonal carcinoma cells. These results revealed that expression of Zeb1 decreased during the differentiation of ESCs, and Lin28a and EMT processes can be regulated by Zeb1, which need to be verified in the future studies.

## 1 Introduction

Zinc finger E-box-binding homeobox 1 (Zeb1), a member of the ZEB family, is a nuclear transcription factor [1,2]. Current studies have shown that Zeb1 can regulate the epithelial-mesenchymal transition (EMT) of cells, mainly promoting the cell EMT process by inhibiting the expression of E-cadherin [3,4]. Most embryonic tissue with developmental arrest or deformity was accompanied by the loss of Zeb1 [1]. Subsequently, the positive or negative regulation of Zeb1 in the directional lineage differentiation of embryonic stem cells (ESCs) has become a research focus. Previous study finds Zeb1 promoter is maintained in a bivalent chromatin configuration in ESC, so that Zeb1 promoter can respond readily to microenvironmental signals, such as TGF-β. In response to the signals, Zeb1 promoter converts from a bivalent to active chromatin configuration and Zeb1 transcription increases [5,6].

As a member of the family of stem cells, ESC under *in vitro* culture can be induced to differentiate into various tissue cells [7]. ESC differentiation involves the process of EMT and mesenchymal-epithelial transition (MET). Generally, the differentiation of ESC into the endoderm is known as the EMT process (we induced mouse ESCs [mESCs] to differentiate into mouse embryonal body [(mEB] and GC1 cell *in vitro*), while the directional lineage differentiation of the endoderm is the MET process [8]. Zeb1, as an EMT-induced factor can promote cell EMT [1]. However, in the process of cell differentiation of MET, changes in Zeb1 expression are not yet clear, and the role of Zeb1 in different lineages of tissue and cell development is still controversial.

Therefore, Zeb1 might play a significant role in the process of ESC differentiation. In the present study, we used mESCs as the subject and induced mESC to differentiate into mEB cell and GC1 cell *in vitro* to explore the functional role of Zeb1 in the processes of cell EMT and MET.

## 2 Materials and methods

### 2.1 Cell culture and transfection

Mouse ESCs (D3) were cultured in mouse ESC media (LIF/serum), which comprise DMEM containing 15% FBS, 100 mM Minimum Essential Medium with Non-Essential Amino Acids (Life Technologies), and 1,000 µ/mL mouse leukemia inhibitory factor (mLIF) (Millipore, USA) with 50 mM 2-mercaptoethanol (Life Technologies). For the EB formation, mESCs were harvested by trypsinization and transferred to bacterial culture dishes in ES medium without LIF. Mouse embryonal carcinoma cell lines (P19) were cultured in DMEM with 1% l-glutamine (Life Technologies) supplemented with 10% FBS. Cells were cultured in a standard humidified incubator at 37°C in a 5% CO_2_ atmosphere.

Zeb1 siRNA was purchased from GenePharma Bio, Inc. (Shanghai, China). The sequence is as this: 5′-GACCAGAACAGUGUUCCAUGUUUAA-3′. As a negative control, the siRNA sequence is 5′-UAUAGCUUAGUUCGUAACC-3′. The cells were incubated with Zeb1 siRNA/negative control siRNA using TurboFect™ siRNA Transfection Reagent (Life Technologies) according to the manufacturer’s protocol. When the optimal confluency for adherent cells is 70–90%, transfection is performed. The transfection reagent/siRNA mixture is added into the cells and incubated at 37°C. Cells were harvested 24–48 h post-transfection for assay.

### 2.2 Total RNA extraction and reverse transcription

Total RNA was extracted from the cells using TRIzol reagent (TaKaRa Bio, Inc., Otsu, Japan) according to the manufacturer’s protocol. 10 µg total RNA was converted to cDNA using the PrimeScript^TM^ RT reagent Kit with gDNA Eraser (TaKaRa Bio, Inc.) according to the manufacturer’s protocol.

### 2.3 qRT-PCR

qRT-PCR was performed using SYBR^®^ Premix Ex Taq^TM^ (TaKaRa Bio, Inc.). Experiments were performed in triplicate. The procedures used are described elsewhere (14). The relative expression was calculated using the Applied Biosystems comparative CT (2^−ΔΔCT^) method. Experiments were repeated in triplicate. Primer sets used for qRT-PCR are listed in Table 1.

**Table 1:** Primers used in the research

Gene	Primer set
Zeb1	Forward: 5′- ACCGCCGTCATTTATCCTGAG -3′
	Reverse: 5′- CATCTGGTGTTCCGTTTTCATCA -3′
GAPDH	Forward: 5′-AGGTCGGTGTGAACGGATTTG-3′
	Reverse: 5′-TGTAGACCATGTAGTTGAGGTCA-3′

### 2.4 Immunofluorescent assay

For immunofluorescent staining, the cells were plated on coverslips and fixed with 4% paraformaldehyde at room temperature for 30 min, permeabilized in 0.1% Triton for 15 min, blocked by 5% albumin from bovine serum (BSA) at 37°C for 30 min, incubated with Zeb1 (NBP1-05987, diluted 1:100 with PBS; Novus, USA) at 37°C for 2 h, and stained with an anti-rabbit-Alexa Fluor 594 antibody (diluted 1:500; Invitrogen, Carlsbad, CA, USA) at 37°C for 1 h. Cell nuclei were stained with Hoechst 33342 (diluted 1:2,000) for 5 min. The images were collected by fluorescence microscopy (Zeiss LSM710, Germany). Experiments were repeated in triplicate.

### 2.5 Western blotting

Total cell protein lysates were extracted by RIPA lysis buffer (Thermo Fisher Scientific, Rockford, USA) and boiled at 98°C for 10 min, then separated by 10% SDS-PAGE at 100 V for 2.5 h and then were sequentially transferred to PVDF membranes (Millipore). After incubation with specific antibodies overnight at 4°C, membranes were washed with TBST buffer and incubated with secondary antibodies for 1 h at room temperature. Western blotting was performed using antibodies specific for Zeb1 (NBP1-05987), Lin28a (ab124765), E-cadherin (CST3195), Vimentin (CST5741), and β-actin (ab8337). Experiments were repeated in triplicate.

### 2.6 Transwell assay

P19 cells were harvested after 24 h of transfection with Zeb1-siRNA. Transwell chamber inserts were used in the assay. On the upper chamber, about 40 µL of Matrigel (0.5 mg/mL, Beckman Coulter, Inc., Brea, CA, USA) was spread and then incubated at 37°C for 4 h. Then, 4 × 10^4^ cells were plated on the upper side of the chamber to detect the migratory ability of P19 cells. In the upper side of the Transwell chambers, cells were resuspended in a serum-free medium, while the lower side of the chambers was filled with a completed medium (10% FBS) at the same time. After incubation for 48 h at 37°C in the incubator, cells on the bottom of the inserts were fixed with 4% paraformaldehyde and then stained with 0.1% crystal violet. Cells that had invaded the membrane were photographed and calculated in four randomly selected fields per well. Experiments were repeated in triplicate.

### 2.7 Statistical analysis

All morphometric data were collected blindly. All statistical analyses were performed using SPSS (version 19.0; SPSS, Inc., Chicago, IL, USA). Two-sided *P* values < 0.05 were considered statistically significant. The data are expressed as the mean value ± SD from three independent experiments.

## 3 Results

### 3.1 Expression of Zeb1 in mESC lines

**Figure 1:**
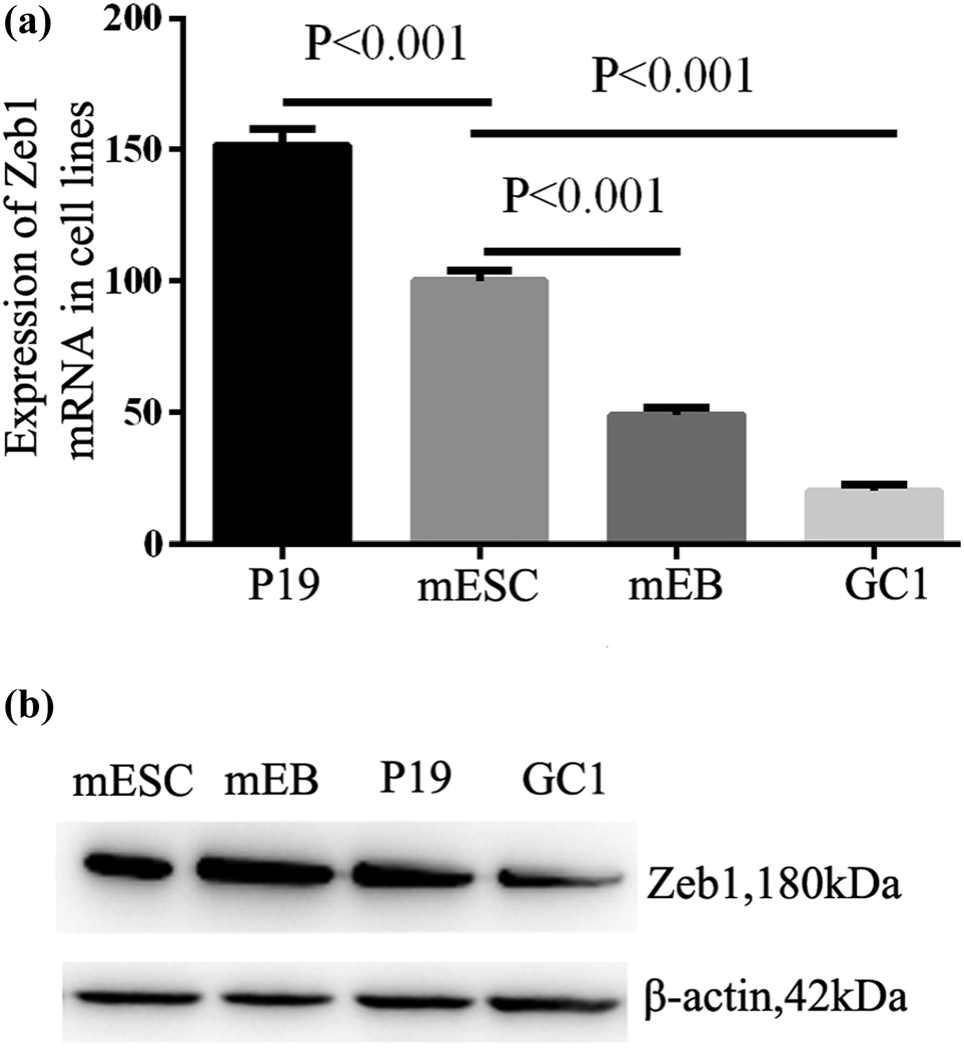
Zeb1 expression in mESCs, mEB, GC1, and P19. (a) Zeb1 mRNA expression detected by real-time qPCR in mESC, mEB, GC1, and P19 (*P* < 0.001, data presented as mean value ± SD). (b) Zeb1 protein expression examined by western blot in mESC, mEB, GC1, and P19. Each experiment was performed in triplicate.

To detect the expression of Zeb1 in ESC lines, we conducted qRT-PCR experiments in three specific mouse cell lines (mESC, GC1, and P19) and mouse embryonal body (mEB). Results showed that Zeb1 existed in all these specific mouse cell lines (Figure 1a). The results indicated that Zeb1 expression significantly decreased during normal differentiation of ESCs (*P* < 0.001), whereas the expression level of Zeb1 in P19 embryonic cancer cells is significantly higher than that in mESCs (*P* < 0.001).

**Figure 2:**
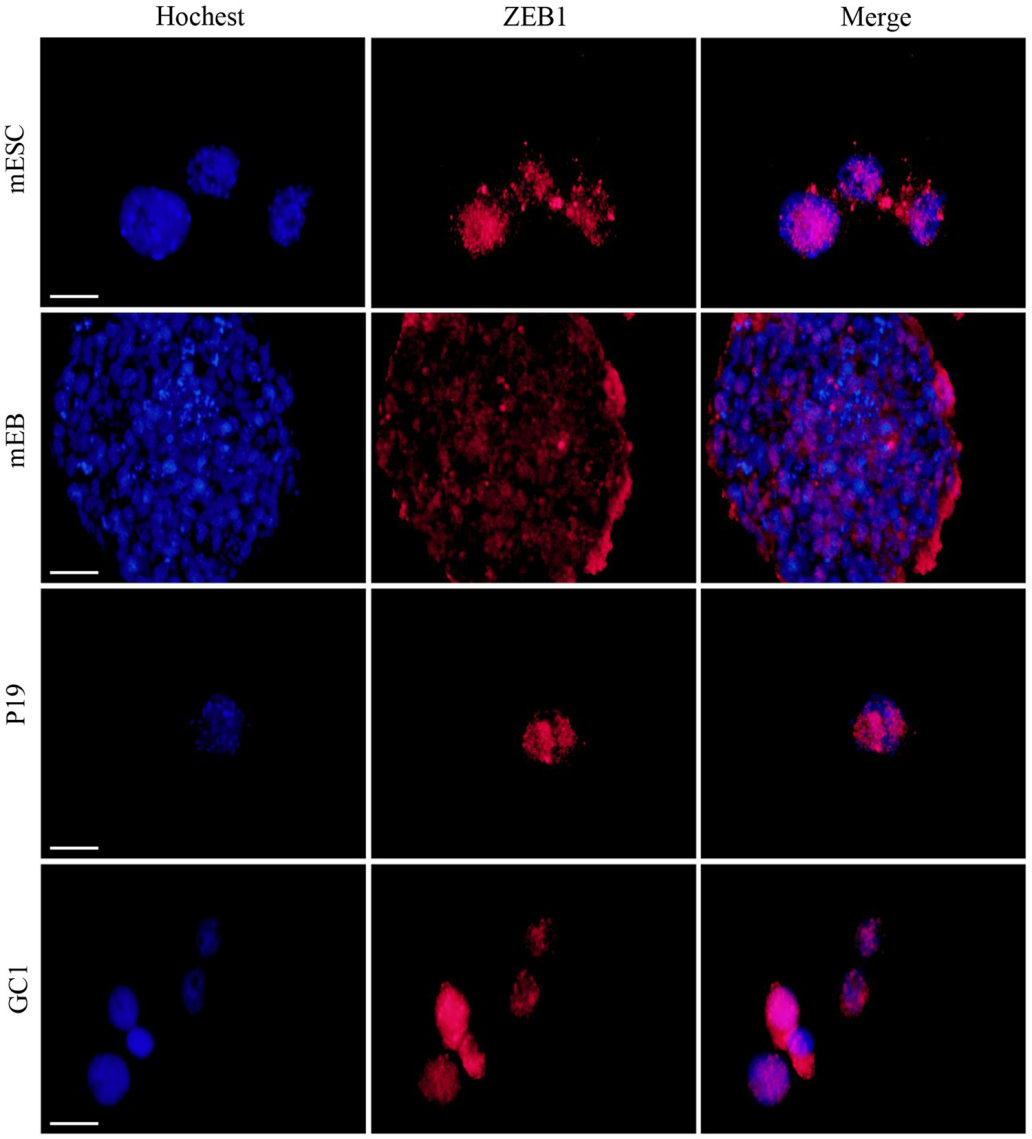
Location of Zeb1 proteins in mEC lines (mESC, mEB, GC1, and P19). Immunofluorescence analysis was used to determine localization of Zeb1 in mESC, mEB, GC1, and P19 using a Zeb1 antibody (red). Cell nuclei were labeled with Hoechst 33342 (blue). The scale for the measurement bar is 50 μm.

Western blot analysis of mESCs, mEB, GC1, and P19 cell proteins indicated that Zeb1 expression significantly differed from that of mRNA. The expression of Zeb1 in embryo cancer cell line (P19) was significantly higher than that of normal adult cell line (GC1, Figure 1b). However, there is no significant difference among mESCs, mEB, and P19. We also used immunofluorescence to detect the location of Zeb1 in mES, mEB, GC1, and P19. The results revealed that Zeb1 protein was mainly located in the nucleus (Figure 2), and previously published studies also showed that Zeb1 was mainly located in the nucleus of mouse neuroblastoma cells [9] and human osteosarcoma cells (Figure S1, obtained from the Human Protein Atlas project resource, www.proteinatlas.org).

### 3.2 Zeb1 inhibited the expression of Lin28a mRNA in mESCs, mEB, GC1, and P19

**Figure 3:**
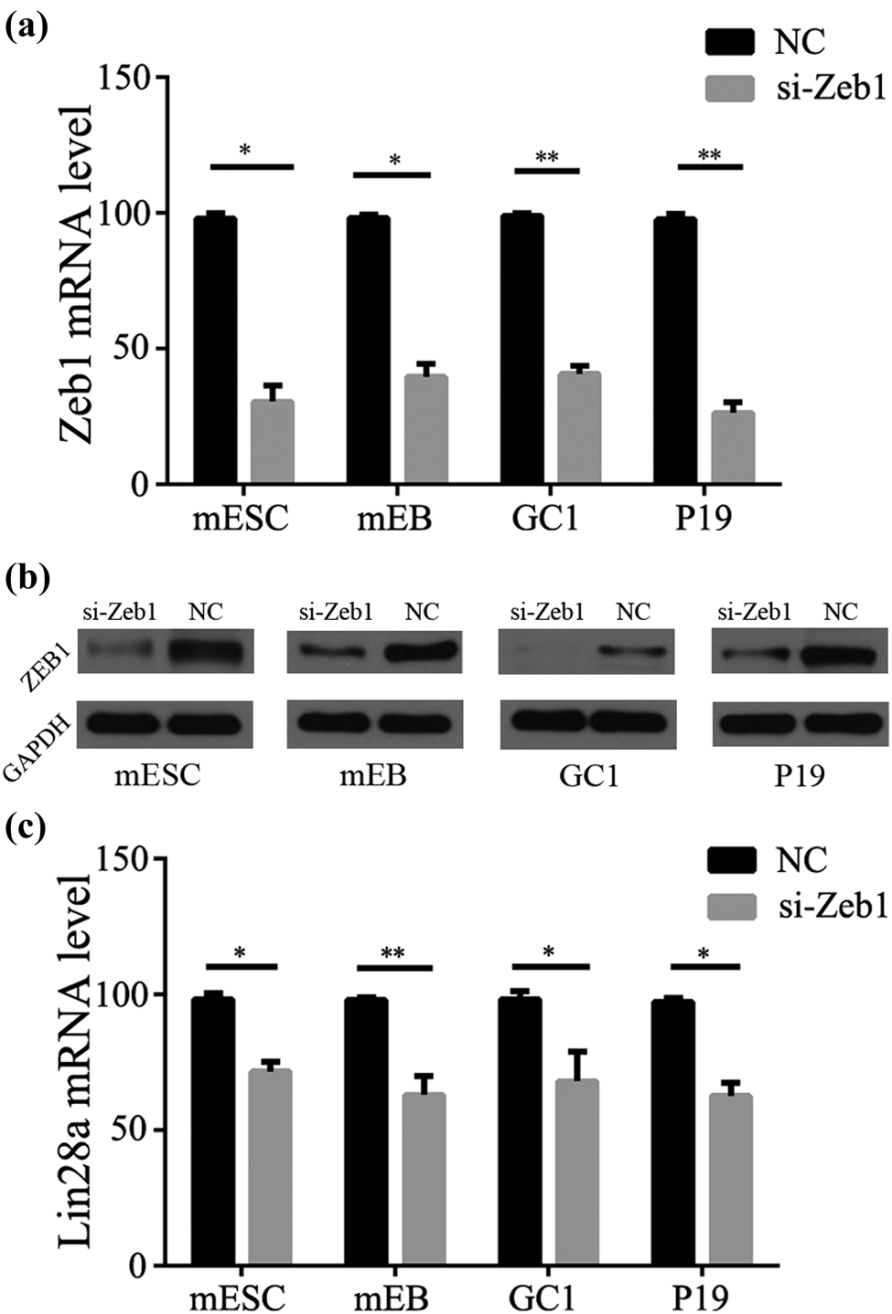
Lin28a expression decreased after transfection with Zeb1 siRNA in mESCs, mEB, GC1, and P19. After Zeb1 knockdown in mESCs, mEB, GC1, and P19 Zeb1, mRNA (a) and protein (b) expression decreased (*P < 0.05, *n* = 3). (c) After Zeb1 knockdown in mESCs, mEB, GC1, and P19, Lin28a mRNA expression decreased (*P < 0.05, **P < 0.01, *n* = 3).

LIN28a is not only a cytoplasm protein but also a nuclear shuttle protein [10]. We observed the expression of Lin28a and EMT- related proteins after the knockdown of Zeb1. As is known to all, Lin28a plays an important role in the embryonic development and stem cells differentiation, and the EMT-stemness model showed that the expression of Lin28a and Zeb1 were positively correlated in the same state of cell types [11]. Thus, the expression of Lin28a protein was detected after the knockdown of Zeb1 in ESCs, mEB, GC1, and P19. According to our results, Lin28a content decreased in all these cell lines after knockdown of Zeb1 (26.55, 35.35, 32.31, and 35.39% in mESCs, mEB, GC1, and P19 cells, respectively) (Figure 3, *P < 0.05, **P < 0.01).

### 3.3 Zeb1 controlled the expression of Lin28a and EMT-related proteins in mESCs, mEB, GC1, and P19 cell lines

**Figure 4:**
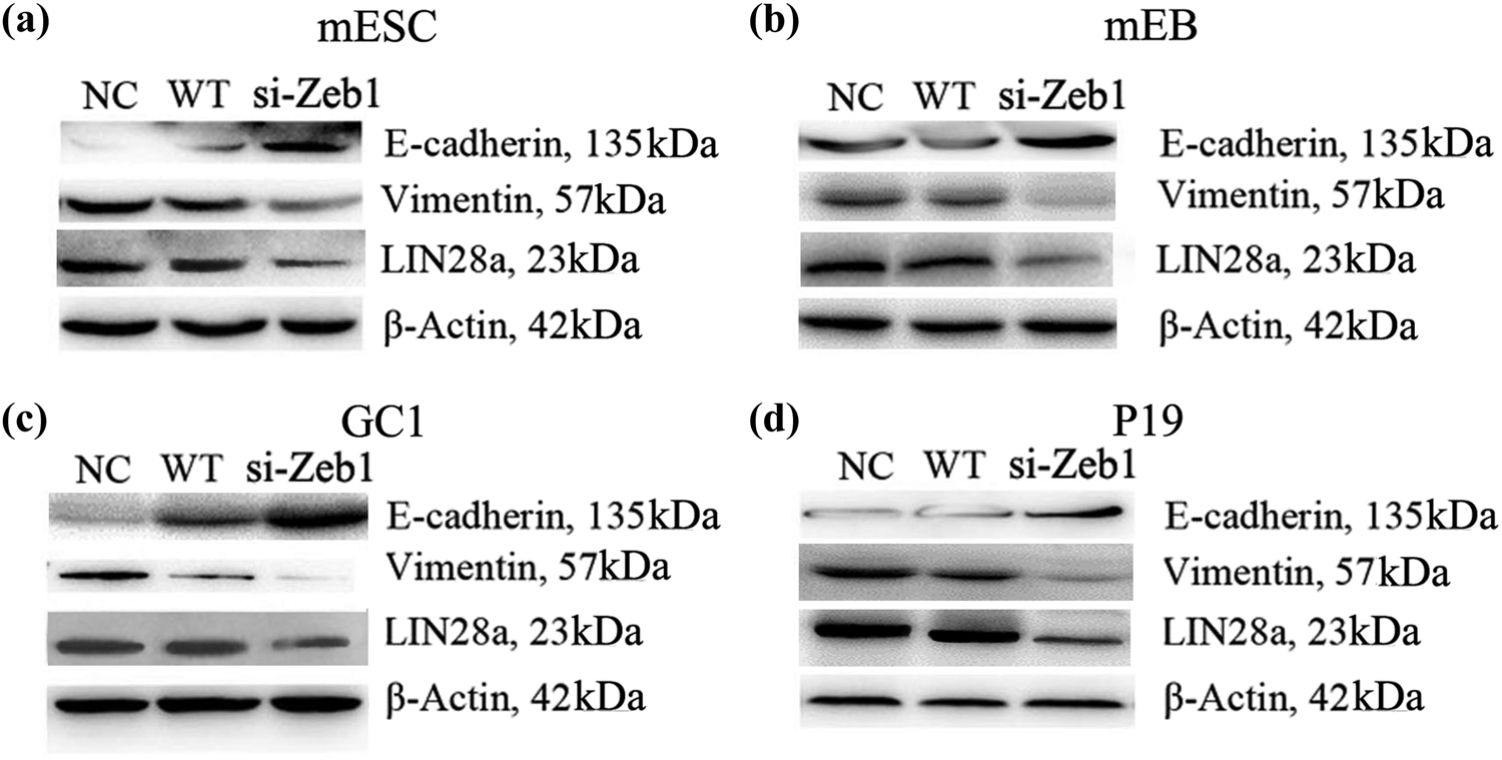
Lin28a and ETM-related protein expression changed after the knockdown of Zeb1 in mESCs, mEB, GC1, and P19. Western blotting demonstrated that E-cadherin protein expression increased and that Lin28a and Vimentin protein expression decreased in Zeb1-siRNA transfected mESC (panel a), mEB (panel b), GC1 (panel c), and P19 (panel d) cells. Each experiment was performed in triplicate. Abbreviations: NC, negative control; siRNA, small interfering RNA; and WT, wild-type.

After transfection of Zeb1 siRNA in the cell group, the expressions of LIN28a and EMT-related proteins (E-cadherin and Vimentin) were detected by Western Blot. After the knockdown of Zeb1, Lin28a and Vimentin protein expressions were decreased, while the expression of E-cadherin was increased during normal differentiation procedure, which can also be observed in P19 cells (Figure 4). Thus, the expression of Lin28a could be inhibited when Zeb1 was knocked down, and cell EMT was inhibited too. However, how Zeb1 mediates Lin28a expression remains to be discovered.

### 3.4 Zeb1 promoted cell invasion of mouse embryonal carcinoma cells through EMT process

**Figure 5:**
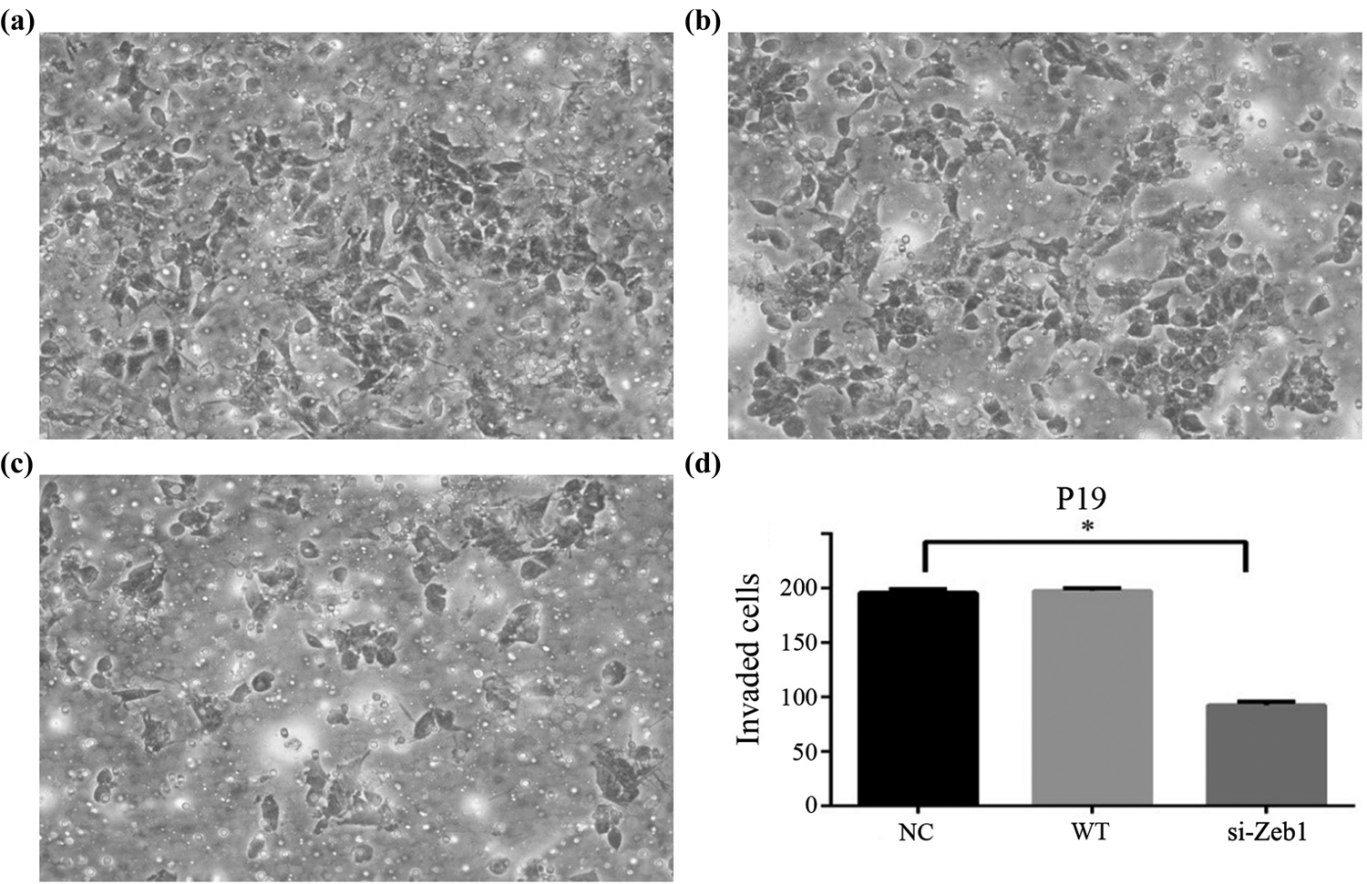
Invasive ability of cells was measured by the Transwell assay. Negative control group (panel a). Wild-type group (panel b). si-Zeb1 group (panel c). Knockdown of Zeb1 decreased embryonal carcinoma cells invasion compared with NC and WT (panel d) (*P < 0.05, data expressed as mean value ± SE). Each experiment was performed in triplicate.

Abnormal differentiation of mouse ESCs developed into embryonal carcinoma. In order to determine the effect of Zeb1, the cell line was subjected to Zeb1 knockdown and cell invasion experiment in this study. When Zeb1 was knocked down, mouse embryonal carcinoma cell lines (P19) exhibited a 51.23 and 49.85% reduction in invasion capacity compared with the negative control and wild type, respectively (Figure 5).

## 4 Discussion

Previous studies reported that Lin28a/let-7 and miR-200/Zeb loop systems regulated pluripotency and malignant transformation of stem cells [12,13,14]. In recent years, abnormal expression of Zeb1 has been found in many tissues and can affect the incidences of fetal developmental arrest and deformity [15,16,17]. In the present study, it is reported that the expression of Lin28a is positively correlated with Zeb1 expression, and knockdown of Zeb1 can decrease expression of Lin28a. Besides the Lin28a/let-7 and miR-200/Zeb loop, previous studies also report that mir-200 can inhibit the protein expression of Lin28a, and let-7 can also inhibit Zeb1 [11,13,18]. In ESC lines, after knockdown of Zeb1, RT-qPCR and western blot results showed that the expression of Lin28a was significantly decreased compared with that of the control group. Thus, to some extent, Zeb1 could regulate the expression of Lin28a.

We demonstrated that Zeb1 mRNA expression levels decreased during normal differentiation of mESCs. The expression levels of Zeb1 in mEB and epithelial cell GC1 were 49.58 and 20.16% of the mESC cell lines. However, the expression level in P19 was about 7.52 times of that in GC1 cell lines. Western blots indicated that Zeb1 expression did not significantly vary among mESCs, mEB, and P19. A possible reason is that the translation of Zeb1 mRNA to protein is limited by enzyme and substrate, which affect the upper limit of Zeb1. It can be inferred that during MET differentiation of ESCs, the expression level of Zeb1 mRNA decreases, while the decrease in Zeb1 protein occurs later than mRNA. However, the expression of Zeb1 in P19 cell lines is significantly higher when compared with GC1 cell line. This reveals Zeb1 is positively regulated by abnormal differentiation of ESCs and tumors. According to immunofluorescence results for mESCs, mEB, GC1, and P19, Zeb1 protein was mainly expressed in terms of its physical location within the nucleus, while Lin28a was mainly expressed as and within the nucleus shuttle protein, which provided the basis for the following study.

Further experiments showed that knockdown of Zeb1 suppressed cell EMT and the invasive ability of embryonic cancer cell lines. In addition, the expression level of stemness factor Lin28a is also suppressed by the knockdown of Zeb1.

Most previous research about Zeb1 described its promoting role in tumorigenesis. However, few studies have focused on the expression and function of Zeb1 in ESC differentiation and canceration. ESC is a kind of cell with unlimited proliferation, self-renewal, and multidirectional differentiation in *in vitro* culture [19]. In the present study, we found that the expression of Zeb1 decreased gradually with the establishment of *in vitro* differentiation system on ESCs, the EBs, and spermatogonial cells. Contrastingly, Zeb1 expression increased significantly in mouse embryonal carcinoma cells. In the present study, it is showed that knockdown of Zeb1 inhibited embryonal carcinoma P19 cell invasion. Previous reports have shown that non-cancer stem cells can be transformed into cancer stem cells when the transcription of Zeb1 increases [20,21]. Therefore, Zeb1 has the potential effect of promoting the canceration of non-cancer stem cells. Based on previous studies and our own results, it can be speculated that the expression of Zeb1 decreased gradually during mESC differentiation. And Zeb1 may play a crucial role in the regulation of differentiation of stem cells. However, the specific function of Zeb1 in cell differentiation needs more experiments to explore. Further, our results also supported that Zeb1 played an important role in regulating the invasive ability of mouse embryonal carcinoma cells.

Except for embryonal carcinoma cells, Cencioni et al. found that mouse ESCs expressed endothelial nitric oxide synthase (eNOS) and endogenously synthesized NO, which in turn inhibited Zeb1 protein and enhanced the expression of microRNA-200 [22]. The present research showed decreased expression of Zeb1 induced the E-cadherin increase. Consequently, stem cells differentiated and promoted MET and transformed to the fate of epithelial cells. Moreover, in human ESCs, findings also indicated that Zeb1 played an important role during neural differentiation in supporting our findings [6]. Thus, Zeb1 existed in multiple species and participated in many cell processes. Recent studies have found that EMT can induce mitochondrial fusion through the Micro200c-PGC1α-MFN1 pathway, activate mitochondrial fusion protein MFN1, and in consequence, promote stem cell self-renewal and maintain the stem cell pool [23]. These results suggested that activation of Zeb1 can promote the activity of the EMT process pathway, induce mitochondrial fusion protein MFN1 expression, and cause mitochondrial dynamic changes.

This research analyzed the expression of Zeb1 both in mRNA and protein levels, as well as the location of Zeb1 protein. We also found that Zeb1 could promote the invasive ability of mouse embryonal carcinoma cells in mESCs differentiation model. Future studies will focus on the role of Zeb1 in mESCs differentiation. It will be assessed whether Zeb1 can affect mESCs differentiation and how the Zeb1-Lin28a pathway induces EMT and differentiation of stem cells.

## Supplementary Material

Supplementary Figure
