# Supplementary material for: Expression of Zeb1 in the differentiation of mouse embryonic stem cell
Source: Open Life Sci. 2022 May 9;17(1):455–62. doi: 10.1515/biol-2022-0042 (PMC9087876; doi:10.1515/biol-2022-0042)
Supplement: Supplementary Figure [file biol-2022-0042-sm.pdf]

Supplementary material

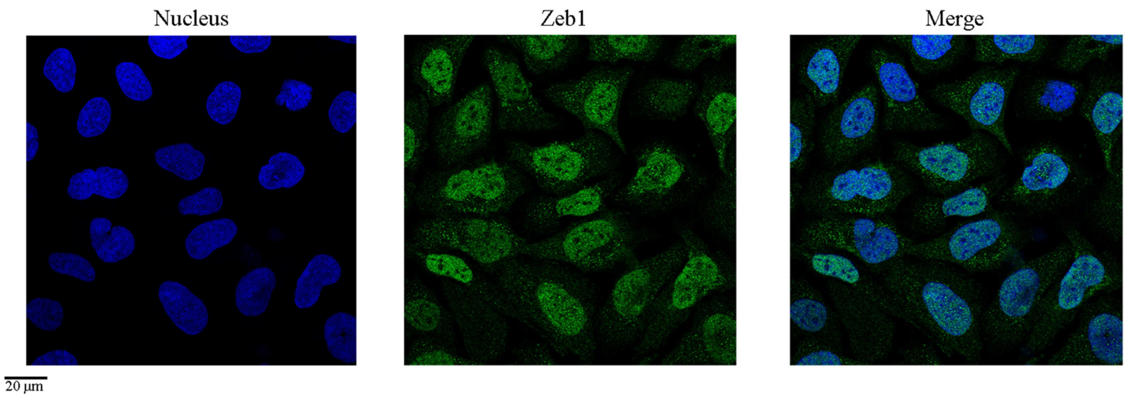

Figure S1: Zeb1 located mainly in cell Nucleus in human U-2 OS cells. Images obtained from the Human Protein Atlas. Scale bar: 20 µm.
